# Supplementary material for: The Potential Role of Fecal Microbiota Transplant in the Reversal or Stabilization of Multiple Sclerosis Symptoms: A Literature Review on Efficacy and Safety
Source: Microorganisms. 2023 Nov 22;11(12):2840. doi: 10.3390/microorganisms11122840 (PMC10745313; doi:10.3390/microorganisms11122840)
Supplement: Supplementary file 1 [file microorganisms-11-02840-s001.zip › microorganisms-2629203-supplementary.pdf]

microorganisms

The Potential Role of Fecal Microbiota Transplant in the Reversal or Stabilization of Multiple Sclerosis Symptoms: A Literature Review on Efficacy and Safety

Tooba Laeeq [1,*], Tahne Vongsavath [1], Kyaw Min Tun [1] and Annie S. Hong [2]

[1] Department of Internal Medicine, University of Nevada, Las Vegas, NV 89154, USA
[2] Department of Gastroenterology, University of Nevada, Las Vegas, NV 89154, USA
* Correspondence: tooba.laeeq@unlv.edu

Supplemental Table S1. Search terms utilized in data extraction.

Search terms utilized in data extraction	
1. Fecal Microbiota transplant	Fecal Microbiome Transplantation OR Intestinal Microbiota Transfers OR Donor Feces Infusions OR Microbiome Transfer Intestinal OR Transplantations Intestinal Microbiome Transplantations OR Fecal Microbiota OR Intestinal Microbiome OR, Fecal Microbiota Transplantation OR Fecal Microbiota Transfer OR Intestinal Microbiome Transfers OR Microbiome Transplantations OR Fecal Infusion OR Donor Feces Transfer, Intestinal Microbiota OR Microbiota Transfer OR Fecal Transfers OR Intestinal Microbiome Transplants OR Intestinal Microbiota Transplantations OR Fecal Microbiome Transplantation OR Microbiota Transfers OR, Intestinal Microbiome Transplants OR Fecal Microbiota Transplant OR Donor Feces Infusions OR Intestinal Microbiota Transplantations OR Fecal Microbiota Transplantations OR Intestinal Microbiota Transplantation OR Intestinal Microbiome Transplants OR Intestinal Microbiome Transplants OR Intestinal Microbiome Transfer OR Fecal Microbiota Transfer OR Intestinal Microbiota Transplantations OR Intestinal Microbiome Transplantation OR Fecal Microbiota Transplantation OR Intestinal Microbiome Transfer OR, Intestinal Microbiome Transplantations OR Intestinal Microbiota Transplantation OR Fecal Microbiome Transplantations OR Fecal Microbiota Transplants OR Fecal Microbiota Transplant OR Fecal Microbiota Transplantations OR Fecal Microbiota Transfers OR Intestinal Microbiome Transplantation OR Fecal Microbiota Transfers OR Donor Feces Infusion OR Intestinal Microbiota Transfers OR Intestinal Microbiota Transplantations OR Intestinal Microbiome Transfer OR Intestinal Microbiota Transplant OR Intestinal Microbiota Transfer OR Intestinal Microbiome Transplant OR Fecal Microbiota Transplantations OR Intestinal Microbiome Transplantations OR Fecal Microbiome Transplantation OR Intestinal Microbiota Transplants OR Fecal Microbiome Transplantation OR Donor Feces Infusions OR Intestinal Microbiota Transplants OR Intestinal Microbiome Transfers OR, Intestinal Microbiota Transfer OR, Donor Feces Infusion OR Intestinal Microbiota Transplantation OR Intestinal Microbiome Transplant OR Intestinal Microbiota Transplant OR Intestinal Microbiota Transplant OR Fecal Microbiota Transplant OR Fecal Microbiota Transplants OR Fecal Microbiota Transfers OR Fecal Transplant OR Fecal Transplantation OR Fecal Transplants
	AND

0.	Multiple Sclerosis	Multiple Sclerosis, Acute Fulminating OR Sclerosis, Multiple OR Disseminated Sclerosis OR Sclerosis, Disseminated OR MS (Multiple Sclerosis)

Supplemental Table S2. Quality assessment scores for case studies.

Author/Year	Study Design	Tool	Score
Kait 2022	Randomized controlled trial	RoB2	
Engen 2020	Case study	NIH tool	7 (Good)
Makkawi 2017	Case study	NIH scale	5 (Good)
Thomas Brody, 2011	Case series	NIH scale	7 (Good)
Victor Garcia-Rodriguez 2020	Case study	NIH scale	6 (Good)

Supplemental Table S3. Quality assessment in detail for case series and case reports using the NIH scale.

	NIH scale	Engen	Brody	Rodri-guez	Makkawi
1.	Was the study question or objective clearly stated?	Yes	Yes	Yes	Yes
2.	Was the study population clearly and fully described including a case definition?	Yes	Yes	No	Yes
3.	Were the cases consecutive?	No	No	No	No
4.	Were the subjects comparable?	No	Yes	No	No
5.	Were the interventions clearly described?	Yes	Yes	Yes	Yes
6.	Were the outcomes measured clearly defined, valid, reliable, and implemented consistently across all study participants?	Yes	Yes	Yes	Yes
7.	Was the length of the follow-up adequate?	Yes	Yes	Yes	Yes
8.	Were the statistical methods well described?	Yes	No	Yes	No
9.	Were the results well described?	Yes	Yes	Yes	No
	Total number of yes	7	7	6	5
	Quality (7–9: Good, 4–6: fair, 1–3: poor)	Good	Good	Fair	Fair

Supplemental Table S4. Quality assessment for randomized controlled trial.

Domain	Predicted direction of bias
Risk of bias arising from randomization process	Low
Risk of bias due to deviations from the intended interventions	Unclear
Missing outcome data	Low
Risk of bias in measurement of outcome	Low
Risk of bias in selection of the reported result	Low
